# Diagnosis of Rare Diseases: a scoping review of clinical decision support systems

**DOI:** 10.1186/s13023-020-01536-z

**Published:** 2020-09-24

**Authors:** Jannik Schaaf, Martin Sedlmayr, Johanna Schaefer, Holger Storf

**Affiliations:** 1 Medical Informatics Group (MIG), University Hospital Frankfurt, Frankfurt, Germany; 2 Institute for Medical Informatics and Biometry, Carl Gustav Carus Faculty of Medicine Technische Universität Dresden, Dresden, Germany

**Keywords:** Rare diseases, Computer-assisted diagnosis, Clinical decision support systems

## Abstract

**Background:**

Rare Diseases (RDs), which are defined as diseases affecting no more than 5 out of 10,000 people, are often severe, chronic and life-threatening. A main problem is the delay in diagnosing RDs. Clinical decision support systems (CDSSs) for RDs are software systems to support clinicians in the diagnosis of patients with RDs. Due to their clinical importance, we conducted a scoping review to determine which CDSSs are available to support the diagnosis of RDs patients, whether the CDSSs are available to be used by clinicians and which functionalities and data are used to provide decision support.

**Methods:**

We searched PubMed for CDSSs in RDs published between December 16, 2008 and December 16, 2018. Only English articles, original peer reviewed journals and conference papers describing a clinical prototype or a routine use of CDSSs were included. For data charting, we used the data items “Objective and background of the publication/project”, “System or project name”, “Functionality”, “Type of clinical data”, “Rare Diseases covered”, “Development status”, “System availability”, “Data entry and integration”, “Last software update” and “Clinical usage”.

**Results:**

The search identified 636 articles. After title and abstracting screening, as well as assessing the eligibility criteria for full-text screening, 22 articles describing 19 different CDSSs were identified. Three types of CDSSs were classified: “Analysis or comparison of genetic and phenotypic data,” “machine learning” and “information retrieval”. Twelve of nineteen CDSSs use phenotypic and genetic data, followed by clinical data, literature databases and patient questionnaires. Fourteen of nineteen CDSSs are fully developed systems and therefore publicly available. Data can be entered or uploaded manually in six CDSSs, whereas for four CDSSs no information for data integration was available. Only seven CDSSs allow further ways of data integration. thirteen CDSS do not provide information about clinical usage.

**Conclusions:**

Different CDSS for various purposes are available, yet clinicians have to determine which is best for their patient. To allow a more precise usage, future research has to focus on CDSSs RDs data integration, clinical usage and updating clinical knowledge. It remains interesting which of the CDSSs will be used and maintained in the future.

## Background

In the European Union (EU), a disease is declared as “rare “if no more than 5 out of 10,000 people are affected [1]. It is estimated that about 7000 different rare diseases (RDs) exist. According to the World Health Organization (WHO), about 400 million people are affected [2]. Many RDs are severe, chronic and life-threatening [3, 4]. 80% of RDs are of genetic origin and pre-dominantly affect children [5–9]. For instance cystic fibrosis as a rare lung disease occurs in the first years of the childhood and is associated with an average life expectancy of 40 years [10]. Other RDs like amyotrophic lateral sclerosis, a degenerative disease of the central and peripheral nervous system, can occur later in life and lead to death within a few years [11]. A big challenge in the management of RDs is finding the right diagnosis. Patients with RDs are sometimes diagnosed too late or not at all. They report many years of a diagnosis odyssey [4].

In the past, several clinical decision support systems (CDSSs) have been developed to support clinicians in finding the right diagnosis for patients with RDs. According to Hunt et al., a CDSS is defined as a system that supports clinical decision-making by comparing characteristics of patients to a knowledge base and collecting and displaying the results [12]. We refer to any system matching this definition as “specific CDSS”. Every other system that a physician might use for decisions, but which does not actively give recommendations based on patient characteristics, is called an “implicit CDSS”.

The information of CDSS is very limited and only two reviews about software for diagnosis support in RDs are currently available. Mueller et al. [13] present an overview of software that can be used to support the diagnosis of RDs. Their article includes different types of software and databases that match both our specific and implicit CDSS categories. In addition, only fully developed systems are presented that are (1) available for download and can be installed on one’s own computer or (2) are only useable online. Systems under development, such as research prototypes or tools in clinical evaluation, have not been considered. However, when developing a new CDSS, software developers require information which prototypes are available and which data and functions they use [12]. The second review by Svenstrup et al. gives an overview of web search, social media and data mining approaches for the diagnosis of RDs. However, this article mainly focusses on their own web search engine FindZebra [14]. Despite their importance, we are not aware of any reviews about developments and current systems of specific RDs CDSSs.

Due to the importance of improving the diagnosis of RDs, we conducted a scoping review in order to map the research performed in this area, to reveal gaps in knowledge as well as to give clinicians an overview of the specific CDSSs that are currently available. The need for this research is highlighted by the fact that the support of diagnosis of RDs using software is part of national strategy plans for RDs, e.g. in Germany (National Plan of Action for People with Rare Diseases [15] and the United Kingdom (The UK Strategy for Rare Diseases) [16].

The objectives of the scoping review were to show clinicians as well as software-developers (1) which specific CDSSs are available to support the diagnosis of patients with RDs, (2) which functionalities and data are used within the specific CDSSs, (3) which CDSSs can be used by clinicians directly and (4) how data can be entered or automatically integrated into the specific CDSSs.

## Methods

The reporting of this scoping review complies with PRISMA-ScR (Preferred Reporting Items for Systematic Reviews and Meta-Analyses extension for Scoping Reviews) [17]. We considered 19 out of 22 PRISMA-ScR items (shown in Additional file 1). We created and uploaded a review protocol in Open Science (URL: https://osf.io/de79v). The author JAS drafted the protocol in December 2018, which was approved by all other authors on December 15, 2018. It was last updated after accessing information about the identified CDSS in January 2020. The final protocol was uploaded for publication retrospectively on 01 March 2020.

### Sources of information and search criteria

To identify relevant articles, we searched PubMed. Unpublished literature was not considered. We did not contact the authors of the articles, but checked the reference lists for further sources of evidence. We retrieved articles published over the course of 10 years, from December 16, 2008 to December 16, 2018, to capture as many relevant publications as possible. The final search was conducted on December 16, 2018.

#### Definition process of search terms

The author JAS performed an initial search in PubMed with a combination of the terms “Clinical Decision Support” and “Rare Diseases”. The terms were combined with a logical “AND”. The goal was to identify relevant keywords for a broad search. The results of the search were 165 publications. To obtain keywords, we checked titles and abstracts of the publications to determine whether they described an specific CDSS for RDs and identified five relevant publications [13, 14, 18–20]. Afterwards, we extracted the keywords from these publications in a brainstorming session with all authors and decided which of them were relevant for the search (see Additional file 2 – Part A). Based on the identified keywords, we created a map to establish a relationship between them (see Additional file 2 – Part B-C).

**Fig. 1:**
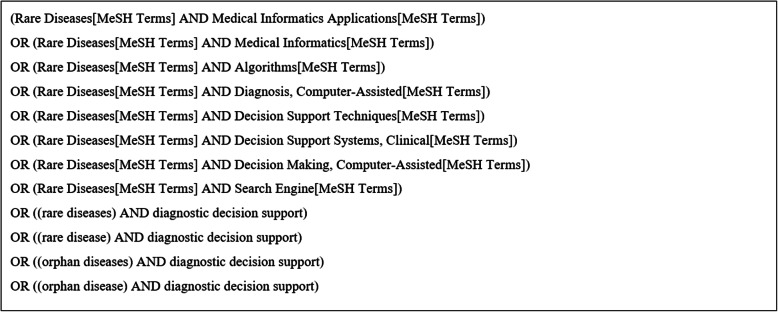
Search Query

In the next step, the identified keywords were tested and mapped to MeSH terms (Medical Subject Heading) by JAS, validated by MS and approved by all authors (described in Additional file 2 – Part D). We also added non-MeSH terms to cover articles which do not appear in the index of MeSH [21]. This led us to our final search terms (shown in Additional file 2 – Part E). We grouped the terms into four groups “A” to “D”. The groups were combined with a logical “AND”. Terms of group “A” and “B” (MeSH terms) and “C and D” (non-MeSH terms) were combined for the search. The search was conducted by JAS with the final query in Fig. 1.

### Inclusion criteria and study selection

We conducted two screening rounds to select publications: A screening based on bibliographic data and a full-text screening. Eligibility criteria are shown in Table 1. JAS created screening forms with Microsoft Word to handle eligibility criteria (Additional file 3). All authors approved the forms.

**Table 1:** Inclusion and exclusion criteria for title and abstract screening and full-text screening

Screening round	Inclusion	Exclusion
Title and abstract screening	The publication is primary research in a peer-reviewed journal or a conference paper.	The publication is of any other publication type. For instance, literature reviews, study protocols, commentaries and editorials were excluded.
Title and abstract screening	The publication has an abstract available.	The publication has no abstract available.
Title and abstract screening	The publication is written in English	The publication is written in any other language than English.
Title and abstract screening	The publication contains primary research or a report of a Clinical Decision Support System for Rare Diseases.	The publication contains any other description of software for Rare Diseases and not a Clinical Decision Support System.
Full-text screening	The publication describes a specific Clinical Decision Support System for Rare Diseases.	The publication describes an implicit Clinical Decision Support System for Rare Diseases.
Full-text screening	The publication describes a clinical prototype or a routine use of a Clinical Decision Support System for Rare Diseases.	The publication describes any other types of Clinical Decision Support Systems implementations (e.g. concepts, software architectures).

In title and abstract screening, we investigated the search result based on the bibliographic data. Publications were included if they contained a peer-reviewed journal or conference paper and an abstract written in English, and if the publication contained primary research of a CDSS in RDs. All other publications were excluded. To test the screening form, we used a random sample of 63 publications (~ 10%). JAS made the decisions on which publications to include, and these decisions were verified by MS. A revision of the form was not necessary, since all authors agreed on the results. After this step, the complete title and abstract screening was performed by JAS and verified by MS. Any disagreements were discussed with all authors and resolved by consensus.

Where available, full-text publications were screened by JAS regarding they describe a specific CDSS for RDs or not. Publications were included if they described a clinical prototype or a routine use of a CDSS for RDs. Publications about any other types of CDSS implementations (e.g. concepts or software architectures), were not considered.

Similar to the abstract and title screening, a screening form for full-text screening was tested by JAS with two (~ 10%) of the remaining publications. We discussed the results amongst all authors and agreed that a revision of the screening form was not necessary. Subsequently, JAS screened all full-text publications available. We considered additional sources using the same eligibility criteria. MS verified the results and any disagreements were resolved by discussion and consensus with all authors. After these two screening rounds, all remaining papers were obtained for data charting.

### Data charting

All authors jointly developed and agreed on a data charting form to determine which data items to extract and guide the author through the data charting process (Additional file 4). We selected the data items based on our research questions. The first version of the data charting form covered six data items. JAS tested the form with five available full-text publications. After the discussion of the pilot test between JAS and MS, we agreed to add four more data items, as of further interest for our research. After the revision of the data charting form, the publications were entered into a spreadsheet by JAS, verified by MS and approved by all authors. The data items are described in Table 2.

**Table 2:** Data items for data charting

Data item	Description
Objective and background of the publication/project	The reader should identify the relevant key message and the objective of the publication or project. This should give the clinician or researcher of this publication an overview of which specific CDSSs for RDs are available and why the project and publication was developed.
System or project name	The “System or project name” is the name of the RDs CDSS, the related project name or the name of the first author.
Functionality	We define “Functionality” as the technology that performs the decision support (e.g. machine learning). The goal is to show the clinicians or researchers how the decision support is derived.
Type of clinical data	“Type of clinical data” indicates whether the CDSS uses clinical routine data (e.g. lab, reports and documentation) or phenotypic and genetic data.
Rare Diseases covered	“Rare Diseases covered” is defined as which diseases are covered by the CDSS.
Development status	“Development status” describes if the CDSS is a clinical prototype or a fully developed system.
System availability	“System availability” describes whether the system is available for download or whether online usage is possible, as well as any access restrictions.
Data entry and integration	“Data integration” describes whether data can be entered manually or transferred automatically into the CDSS (e.g. via file upload or REST-API).
Last software update	“Last software update” reports when the CDSS was last updated to a new software version.
Clinical usage	“Clinical usage” reports if there are any information about CDSS available regarding clinical usage (e.g. amount of patient cases, amount of users, participating hospitals)?

### Summarizing and reporting the results

To present the results, we prepared an overview of all results regarding the data items. Furthermore, we grouped each relevant CDSS according to the data item “Functionality”. We described the background of a CDSS and the data used to perform decision support. We also prepared an overview of the development status of each CDSS and stated whether and how the system is available. Furthermore, we described how data can be entered or integrated into the CDSS and provided information about the latest updated software version. We also provided a short summary including all data items at the end of each section.

## Results

**Fig. 2:**
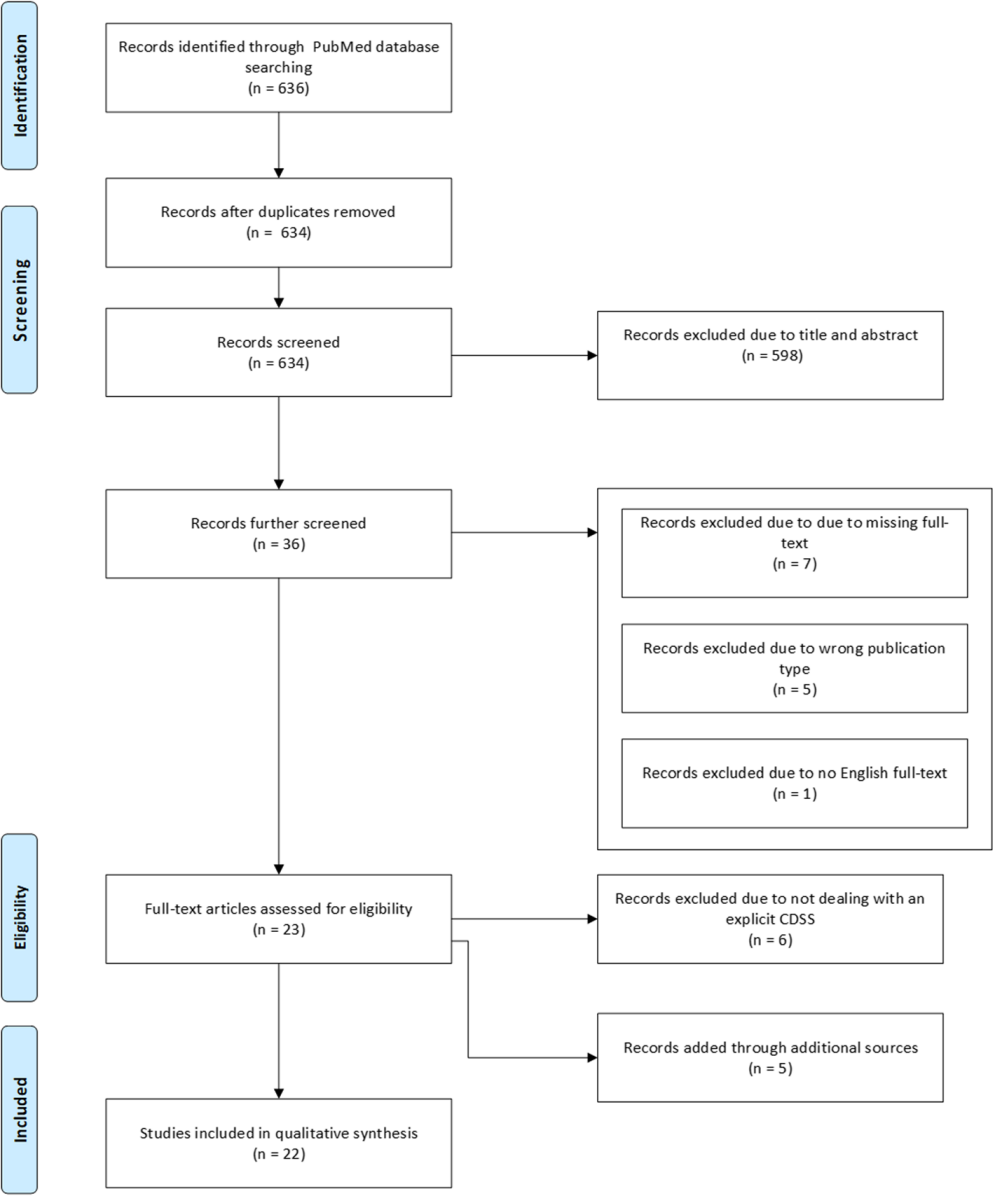
Scoping literature review flowchart

The search identified 636 articles in PubMed (shown in Fig. 2). After removing two duplicates, 634 articles were available for title and abstract screening. In the first screening step, 598 articles were excluded and 36 articles were considered relevant. This number was further reduced due to not accessible full-text of seven articles, wrong publication types in five articles and no full-text in English in one article. After assessing the eligibility of the remaining 23 articles, six articles were excluded because they did not deal with a specific CDSS and were neither in clinical nor in routine use. This resulted in 17 articles and an additional of five articles [22–26] were added after checking the reference list of the publications. A total of 22 articles were available, describing 19 different CDSSs (shown in Additional file 5).

### Overview of the results

Twelve out of nineteen CDSSs use “analysis or comparison of genetic and phenotypic data” as a CDSS functionality [22–25, 27–34]. Three of the CDSSs are based on “machine learning” [20, 26, 35]. Four CDSSs use “information retrieval” [18, 36–38]. The results show that five CDSSs are clinical prototypes [18, 20, 26, 35, 36] whereas 14 are fully developed systems [22–25, 27–34, 37, 38]. Twelve CDSSs use phenotypic and/or genetic data [22–25, 27–34], three use literature databases [18, 37, 38]. Six use clinical data in addition to other data [24, 29, 30, 32, 34] and two use clinical data only [20, 36]. Two CDSSs use patient questionnaires [26, 35]. In 15 of 19 CDSS, all RDs can be included for decision support [18, 22, 23, 27–34, 37, 38]. Only four CDSS are restricted to a group or different RDs [20, 26, 35, 36].

Regarding system availability, five CDSSs are not available for personal use [18, 20, 26, 35, 36], six CDSSs can be used online and free after completing a registration [27, 28, 30, 31, 33, 34]. Furthermore, three CDSS can be used online and free without any registration [22, 29, 37]. One CDSS can be downloaded without any registration [38]. Four CDSSs can be downloaded, but require registration before access is granted [23, 24, 31, 32]. Data can be entered only via forms in four CDSSs [22, 28, 31, 37], whereas six CDSSs additionally allow the upload of data and files [23, 25, 29, 32–34]. We could not find exact information on data entry or integration for four CDSSs [18, 20, 26, 35]. Six CDSSs described further ways of a data integration complying with the REST standard [24, 27, 30, 32–34], whereas data can be integrated with ETL processes in one CDSS [36] and with data upload only in one other CDSS [38].

There is no information about software updates for 13 CDSSs [18, 20, 22, 23, 25–28, 33, 35–38], whereas six CDSS were updated between November 2017 and August 2019 [24, 29–32, 34]. One CDSS provided regular updates, but no information about the release date was provided [24]. Furthermore, we could not find any reporting about the current clinical usage for 13 CDSS [18, 20, 22–26, 28, 29, 31, 35, 37, 38], whereas 6 CDSS provide information [27, 30, 31, 33, 34, 36]. Table 3 shows a comparative overview of the results.

**Table 3:** Results of the charted data items

Data item	Subcategories	Total/Frequency
Functionality	Analysis or comparison of genetic and phenotypic data	12 (63.2%)
	Information retrieval	4 (21.0%)
	Machine learning	3 (15.8%)
Development status	Fully developed systems	14 (73.7%)
	Clinical prototypes	5 (26.3%)
Type of clinical data	Phenotypic and/or genetic data	12 (63.2%)
	Clinical data	8 (42.1%)
	Literature databases	3 (15.8%)
	Patient questionnaires	2 (10.5%)
Rare Diseases covered	All rare diseases	15 (78.8%)
	Neuromuscular rare diseases	1 (5.3%)
	Pulmonary rare diseases	1 (5.3%)
	Rare brain cancer diseases	1 (5.3%)
	Other	1 (5.3%)
System availability	The system can be used online and free, subject to registration	6 (31.6%
	The system is not available for personal use	5 (26.3%)
	The system can be downloaded, subject to registration	4 (21.0%)
	The system can be used online and free, no registration necessary	3 (15.8%)
	The system can be downloaded, no registration necessary	1 (5.3%)
Data entry and integration	Data entry with forms and data upload is possible	6 (31.6%)
	REST-API available	6 (31.6%)
	Data entry is only possible with forms	4 (21.0%)
	No information available	4 (21.0%)
	ETL processes	1 (5.3%)
	Data upload is possible	1 (5.3%)
Last software update	No information available	13 (68.4%)
	Information available	6 (31.6%)
Current clinical usage	No information available	13 (68.4%)
	Information available	6 (31.6%)

### CDSSs using machine learning

Machine learning (ML) allows computer-systems to “learn” from data using statistic methods [39]. CDSSs using ML can be trained using medical data in order to support the decision of a clinician [40]. In this section, we show CDSSs for RDs using ML.

Rother et al. [26], Grigull et al. [35] and Sidiropoulos et al. [20] developed clinical prototypes using ML algorithms for the diagnoses of patients with rare pulmonary diseases, rare neuromuscular diseases and rare cancers. Grigull et al. [35] and Rother et al. [26] focused on rare pulmonary and neuromuscular diseases and used patient-related questionnaires to train ML algorithms. In the results, they achieved a diagnosis rate of 89% respectively 94% [26, 35]. Sidiropoulos et al. [20] developed a real-time decision support system for the diagnosis of rare cancers. The authors used a GPU framework (Graphics Processing Unit) to show a result in real time based on histological clinical data. This allows a faster real-time decision than on a CPU-based system (Central Processing Unit). The system subsequently suggested the correct diagnosis in about 74% of the cases and performed up to 288 times faster than on the CPU [20]. Since all three CDSSs are clinical prototypes, no information about software update, clinical usage, data integration and access for clinicians are available.

#### Summary for clinical usage

Table 4 shows the summary of “CDSSs using machine learning”.

**Table 4:** Summary for clinical usage – CDSSs using machine learning

CDSSs using machine learning	• Rother et al. [26]
	• Grigull et al. [35]
	• Sidiropoulos et al. [20]
Development status and system availability	• All CDSSs are clinical prototypes
	• All CDSSs are currently unavailable for usage
Type of clinical data	• Rother et al., Grigull et al.: Patients questionnaire
	• Sidiropoulos et al.: Clinical data
Rare Diseases covered	• Rother et al.: Pulmonary rare diseases
	• Grigull et al.: Neuromuscular rare diseases
	• Sidiropoulos et al.: Rare brain cancer diseases
Data entry and integration	• No information available
Last software update	• No information available
Current clinical usage	• No information available

### CDSSs using information retrieval

Online databases, like PubMed, are consulted by clinicians to search for case reports of patients. Often, case reports are manually compared to identify similar characteristics of patients. This process is time-consuming and inefficient. With the help of methods like Information retrieval (IR), it is possible to find information, especially in large databases or on the internet [41]. IR includes different techniques to retrieve information based on keywords. For instance, search engines like Google use IR methods [42]. In this review, we show CDSSs using IR which support the diagnosis of RDs.

#### Identify relevant information in databases based on symptoms and phenotypes

FindZebra [37] is a search engine and a fully developed system that allows clinicians to enter symptoms in a search field and find corresponding information in databases. The knowledge base of FindZebra is built on 33,144 documents covering approximately 90% of the RDs listed in the Orphanet database. FindZebra uses ten sources for their dataset, for instance OMIM (Online Mendelian Inheritance in Man) and GARD (Genetic and Rare Diseases Information Center) [37]. OMIM contains descriptions about human genes and their correlation with phenotypes, which are defined in genetics as a set of all visible characteristics of an organism [37]. GARD provides a database about RDs with symptoms, treatments and further research information [37].

To evaluate FindZebra, the authors compared FindZebra with other platforms like PubMed and Google, using 56 search queries with patient symptoms based on expert knowledge. The findings show that FindZebra outperforms Google and PubMed. Especially for queries with a long list of symptoms, FindZebra achieves better results. Google uses an algorithm based on how often a website is visited or linked to other websites. The authors concluded that this would lead to poor results for RDs [37]. However, an information about software updates, clinical usage or further data integration is not provided.

A further CDSS to identify relevant information in a database is the CDSS of Taboada et al. [38]. The authors described a fully developed system which can automatically capture relevant literature data based on phenotypes. Their CDSS uses so-called “text annotation”, a method from the field of Natural Language Processing (NLP), to identify relevant words in a text.

The evaluation of the CDSS was based on a disease “Cerebrotendinous xanthomatosis” (CTX), a rare disorder of bile acid metabolism. The authors extracted 223 abstracts of case reports from PubMed corresponding to CTX. Only the title and relevant parts of the texts were used for annotation. Those were annotated with the Human Phenotype Ontology (HPO) using the software Open Biological and Biomedical Ontologies (OBO) and Bioportal. The HPO describes the correlation between phenotypes and genetic diseases.

The evaluation was measured between the automatic annotation method and a manual annotation of two neurologists, who extracted the relevant phenotypes manually. The authors evaluated the capability to identify the relevant papers for both methods (F-measure). The CDSS achieved an F-measure of 74%, which is significantly lower than the result of the manual method with 88% [38]. The authors concluded that the annotation method could have a high impact on the quality of the results [38].

FindZebra [37] and Taboada et al. [38] both provide fully developed systems, using literature databases, but different ways to find relevant data about RDs in databases. As with FindZebra, no information about software update or clinical usage is available.

#### Using data of electronic health records for the recommendation of Rare diseases

Garcelon et al. [36] developed a clinical prototype to find similar patients to an undiagnosed patient (index patient) in a clinical data warehouse containing about 400,000 patients. The data warehouse is a combination of different sources, e.g. electronic health records (EHR). The similarity is calculated using the Vector Space Model (VSM), by representing patient data in a mathematical vector. The similarity of two patients is measured as the presence or absence of words in the compared patient vectors [36].

Five different rare genetic diseases with 7 to 103 patient cases per disease were used for the evaluation of the CDSS. The authors evaluated its capability to find the patients who were most similar to an undiagnosed patient. Patients were considered to be similar (called true positive similar patients) when they were among the top 30 of the most similar patients and also appeared in the list of diagnosed patients, which was provided by a domain expert. The percentages of index patients, returning at least one true positive similar patient in the list of the top 30 similar patients, were reported as 94% for Lowe Syndrome, 97% for Epidermolysis Bulloas, 86% for Activated PI3K Delta Syndrome, 71% for Dowling Meara and 99% for Rett Syndrome. The average number of patients with the same disease among the top 30 similar patients was 51% [36]. Although the system achieved good results in diagnostics, it cannot be accessed and no information about a software update is available. However, data integration is described with ETL processes.

Shen et al. [18] developed a clinical prototype that uses not only clinical data. The authors merged clinical and literature data. They included clinical data from 13 million unstructured clinical notes on 700,000 patients’ electronic health records limited to described problems and diagnosis. Abstracts from research articles from the SemMedDB were extracted for the literature dataset. SemMedDb is a repository of semantic predications, extracted from titles and abstracts of all PubMed citations. The authors applied HPO and GARD terms to match the representation of both data types, followed by data fusion strategies to include the data into a collaborative filtering model to enable RD recommendation. Data fusion means that different types of data sets are combined into one dataset [18]. The authors used the following data fusion strategies: First, only the patient phenotype information was extracted from the EHR. For the second, the authors combined EHR data with phenotypes and literature. In the third fusion strategy, phenotype-rare disease associations were extracted from literature with the limitation that phenotypes of the literature data were deleted if they did not appear in the EHR data. The authors then evaluated the prediction output for each fusion strategy using a collaborative filtering model to determine which possible combinations provide the best results. This technique is used, for instance, in e-commerce to recommend products to customers based on similar buying preferences of other customers. That this scenario is similar to patients’ phenotypic information. If patients have similar phenotypes, their diseases might also be similar. The results are compared with the actual diagnosis of the patient [18]. The results show that the combination of EHR and literature data did not always lead to the best performance. The authors conclude that this may be due to different approaches and expressions in clinical notes varying from physician to physician [18]. Since the CDSS is a clinical prototype, the system cannot be accessed, no information for data entry and integration is available and neither there are any information about software updates and clinical usage.

#### Summary for clinical usage

Table 5 shows the summary of “CDSSs using information retrieval.”

**Table 5:** Summary for clinical usage – CDSSs using information retrieval

CDSSs using information retrieval	• FindZebra [37]
	• Taboada et al. [38]
	• Garcelon et al. [36]
	• Shen et al. [18]
Development status and system availability	• FindZebra: Fully developed system. The system can be used online and free, no registration necessary
	• Taboada et al.: Fully developed system. The system can be downloaded, no registration necessary
	• Garcelon et al., Shen et al.: Clinical prototype. The system is not available for personal use
Type of clinical data	• Taboada et al., FindZebra.: Literature databases
	• Garcelon et al.: Clinical data
	• Shen et al.: Literature databases and clinical data
Rare Diseases covered	• Taboada et al., Shen et al., FindZebra: All rare diseases
	• Garcelon et al.: Lowe Syndrome, Dystrophic Epidermolysis Bullosa, Activated PI3K delta Syndrome, Rett Syndrome, Dowling Meara
Data entry and integration	• FindZebra: Data entry is only possible with forms
	• Taboada et al.: Data upload is possible
	• Shen et al.: No information available
	• Garcelon et al.: ETL processes
Last software update	• No information available
Clinical usage	• FindZebra: No information available
	• Taboada et al.: No information available
	• Shen et al.,: No information available
	• Garcelon et al.: 400.000 patients included

### CDSSs using analysis or comparison of genetic and phenotypic data

When dealing with complex symptoms of patients with RDs, it is important to identify phenotypes and to combine it with genetic testing to determine the cause of the disease (genotype) [33]. Whole-genome sequencing (WGS) and whole-exome sequencing (WES) provide possibilities to meet these challenges [29]. However, understanding the complexity of the genetic variants which can cause a disease, remains a challenge for clinicians [24]. Different software programs have been developed to tackle these problem. We call these tools “CDSSs using analysis and comparison of genetic and phenotypic data”. To showcase the results, we distinguish between (1) “CDSSs using analysis of genetic and phenotypic data”, which allow the investigation of genetic variants and their correlated phenotypes and (2) “CDSSs using comparison of genetic and phenotypic data”, which enable the identification of similar patients.

#### Investigation of phenotype and genotype correlations

Our review includes Phenopolis [31], GEMINI [24] and GenIO [29], which provide different tools for the investigation of genetic variants. All three of these CDSSs are fully developed systems and can be accessed by clinicians. We show further characteristics in Table 6.

**Table 6:** Summary for clinical usage – CDSSs using analysis of genetic and phenotypic data

CDSSs using analysis of genetic and phenotypic data	• GenIO [29]
	• Phenopolis [31]
	• GEMINI [24]
Development status and system availability	• GenIO: Fully developed system. The system can be used online and free, no registration necessary
	• Phenopolis: Fully developed system. The system can be downloaded, subject to registration
	• GEMINI: Fully developed System. The system can be downloaded, subject to registration
Type of clinical data	• GenIO: Phenotypic, genetic and clinical data
	• Phenopolis: Phenotypic data and genetic data
	• GEMINI: Phenotypic, genetic and clinical data
Rare Diseases covered	• GenIO: All rare diseases
	• Phenopolis: All rare diseases
	• GEMINI: All rare diseases
Data entry and integration	• GenIO: Data entry with forms and data upload is possible
	• Phenopolis: Data entry is only possible with forms
	• GEMINI: Data upload and REST API available
Last software update	• GenIO: Version 1.0, 22nd of November 2017
	• Phenopolis: Version 1.0.2, 12 h of November 2017
	• GEMINI: Version 0.20.1, no date available
Clinical usage	• GenIO: No information available
	• Phenopolis: No information available
	• GEMINI: No information available

Phenopolis [31] provides an open-source web server and different analysis tools like variant filtering and gene prioritization based on phenotypes of a patient using the HPO. Variant filtering allows to identify relevant variants for a diagnosis. With gene prioritization, potentially causative genes can be prioritized. Phenopolis contains 6048 exomes representing the 4,859,971 variants which comprise the data base [31].

GEMINI [24] is a software package which allows researchers to integrate the genetic variations in the Variant Call Format (VCF), a common format for the gene sequence variants. GEMINI provides variant analysis tools for the investigation of variants and a programming interface to customize the data analysis and exploration [24]. Koile et al. developed GenIO [29] a web interface for clinicians and researchers who do not have the necessary skills to annotate, classify and filter variants. GenIO uses the so-called “GenIO pipeline“, which consists of a variant annotation and phenotype processing [29]. At the end of the phenotyping process, a list of genes with matches to the patient’s phenotype is shown [43].

#### Summary for clinical usage

Table 6 shows the summary of “CDSSs using analysis of genetic and phenotypic data”.

#### Finding similar patients and sharing patient cases

The identification of similar patients and the sharing of patient cases is possible with GeneMatcher [27], GeneYenta [28], Phenotips [32], PhenomeCentral [34], MatchMaker Exchange [30], DECIPHER [33], PhenoDB [23] and GenomeConnect [25]. All of these CDSSs are fully developed systems and can be accessed in different ways. They allow clinicians and researchers to find similar patients in a database based on genetic or phenotypic data. We show further details for clinical usage in Table 7.

**Table 7:** Summary for clinical usage – CDSSs using comparison of genetic and phenotypic data

CDSSs using comparison of genetic and phenotypic data	• DECIPHER [33]
	• GeneMatcher [27]
	• GeneYenta [28]
	• Matchmaker Exchange [30]
	• PhenomeCentral [34]
	• PhenoTips [32]
	• Phenomizer [22]
	• PhenoDB [23]
	• GenomeConnect [25]
Development status and system availability	• DECIPHER, GeneMatcher, GeneYenta, Matchmaker Exchange, PhenomeCentral, GenomeConnect: Fully developed system. The system can be used online and free, subject to registration
	• PhenoDB, PhenoTips: Fully developed System. The system can be downloaded, subject to registration
	• Phenomizer: Fully developed system. The system can be used online and free, no registration necessary
Type of clinical data	• DECIPHER, GeneMatcher, PhenoDB, GenomeConnect: Phenotypic and genetic data
	• GeneYenta, Phenomizer: Phenotypic data
	• Matchmaker Exchange, PhenoTips, PhenomeCentral: Phenotypic, genetic and clinical data
Rare Diseases covered	• DECIPHER, GeneMatcher, GeneYenta, Matchmaker Exchange, PhenomeCentral, PhenoTips, Phenomizer, PhenoDB, GenomeConnect: All rare diseases
Data entry and integration	• DECIPHER, PhenomeCentral, PhenoDB, GenomeConnect, PhenoTips: Data entry with forms and data upload is possible
	• GeneMatcher, GeneYenta, Phenomizer: Data entry is possible with forms
	• Matchmaker Exchange, DECIPHER, GeneMatcher, PhenomeCentral, PhenoTips: REST API available
Last software update	• DECIPHER, GeneMatcher, GeneYenta, Phenomizer, PhenoDB, GenomeConnect: No information available
	• Matchmaker Exchange: Version 1.1, 20th of August 2019
	• PhenomeCentral: Version 1.2.0, 14th of August 2019
	• PhenoTips: Version 1.4.7, 17 h of May 2019
Clinical usage	• DECIPHER: 270 centres, 36.000 patient cases
	• GeneMatcher: 8807 users from 90 countries
	• GeneYenta: No information available
	• Phenomizer: No information available
	• PhenoDB: No information available
	• Matchmaker Exchange: Information about connected platforms are available (see https://www.matchmakerexchange.org/statistics.html)
	• PhenomeCentral: 10.000 patient cases
	• PhenoTips: Used in over 60 countries
	• GenomeConnect: No information available

PhenomeCentral, DECIPHER and GeneYenta are connected to the Matchmaker Exchange Project (MME) which connects organizations and projects through a federate network of databases of genotypes and rare phenotypes using a REST API [30]. A REST (Representational State Transfer) API (Application Programming Interface) is a web architecture style and provides the opportunity to shift data to another software system over the internet [44]. The MME enables searches across multiple databases from different platforms by making requests to all databases, e.g. to find similar matches of patients. In order to use MME, a clinician must therefore be part of a participating MME project. MME itself does not provide a user interface, but only connects existing platforms via the MME API [30].

Another CDSS which uses the similarity of phenotypes by not comparing different patient cases is Phenomizer [22]. Phenomizer is a fully developed system and facilitates differential diagnoses by using the HPO for entering phenotypes. The software classifies all diseases listed in OMIM, Orphanet and DECIPHER and uses a semantic similarity metric to measure the similarity between phenotypes and genetic diseases. Several symptoms of a patient can be entered and combined to describe the entire spectrum of a patient’s symptoms. All related diagnoses with their statistical probability are shown to rank the candidate’s disease [22].

#### Summary for clinical usage

Table 7 shows the summary of “CDSSs using comparison of genetic and phenotypic data”. For interested developers, we provide links to the respective REST APIs showing which data can be integrated (Additional file 6).

## Discussion

### Summary of evidence and interpretation

Our scoping review is the first to summarize the evidence of specific CDSSs for the diagnosis support for RDs. We identified 19 CDSSs between 2008 and 2018. Our findings show that most used methods of CDSSs are analysis or comparison of genetic and phenotypic data, followed by information retrieval and machine learning. However, we could not identify many publications considering machine learning, although it plays an increasing role in healthcare [45]. In other medical fields, a higher number of CDSSs can be found. For example, a review found 60 CDSSs for infectious diseases [46], while a systematic review in cardiology identified 331 relevant studies [47]. This might have been caused by the fact that machine learning for RDs is currently a problem due to a lack of data pertaining to RD patients. Garcelon et al. even recommended in their study to focus on other methods for clinical decision support [36].

Since most CDSSs use analysis or comparison of genetic and phenotypic data, this is also the most used data, followed by clinical data, literature databases and patient questionnaires. From the clinician’s point of view, describing phenotypes is a challenging task. Individual patients are often not comprehensively described, for instance when a patient does not report all symptoms. If an anomaly in an individual patient is not described, it does not follow that this anomaly does not exist. The description of the phenotype features also depends on the clinician’s experience. Another problem is that the same phenotype can be caused by multiple genetic defects [29].

Furthermore our review shows, that 15 of 19 CDSS can be used for all different kinds of RDs. Only 4 CDSS are limited to different RDs. However, these CDSS have been assessed with this RDs. Therefore it is unclear whether these CDSS can be used with other diseases.

Our review includes 14 CDSSs that are fully developed systems and can be used by the clinicians directly. All of these systems can be used online or via free download. For some of them, a registration is required. Only five systems are clinical prototypes and cannot be accessed.

### Identified gaps of knowledge

Our findings indicate a lack of CDSSs that allow automatic data integration. Only seven of 19 CDSSs use ETL processes or REST interfaces. However, four studies did not describe how the data can be entered into their CDSS. We conclude this is an essential factor for the acceptance of a CDSS. Redundant data entries into several systems should be avoided [48, 49]. There are studies available specifically dealing with data integration into CDSSs [50–52], which appears to be a major challenge due to the heterogeneity of the information systems used in healthcare [53, 54]. As a possible solution, CDSSs could utilize interoperable standards such as FHIR (Fast Healthcare Interoperability Resources) for data integration [66, 67].

Furthermore, the timeliness of the data in a CDSS is important, and there should be information on when it was last updated and maintained. CDSSs should be updated regularly to integrate a new balance of evidenced-base medicine or end user preferences [55, 56]. For instance, the Roadmap for National Action on Clinical Decision Support of the American Medical Informatics Association recommends that CDSS knowledge bases and methods should be improved continuously [57]. Regarding the timeliness of data in a CDSS, only six CDSSs provide information, e.g. on their websites. These CDSSs were updated between November 2017 and August 2019.

However, since 14 CDSS are fully developed systems, we could not find any information of clinical usage for 13 CDSS. It was not possible to find sufficient and sustainable information. For instance, no evaluation reports, investigating the clinical usage, were available. It remains interesting how often and by how many users CDSS for RDs are used.

### Limitations

This work provides a broad overview of 19 different CDSSs which employ different approaches and functionalities to support the diagnosis of RDs. Not every CDSS could be explained in detail, and each system’s background, medical but also technical aspects are only touched upon to provide an overview. However, this scoping review is a starting point to show clinicians and software developers what is known in the context of CDSSs for RDs. Further studies may take up this review and carry out further investigations.

Furthermore, the literature of this scoping review is up-to-date as of December 2018. We accessed the information of the topicality of the CDSS in January 2020. However, it was not possible to establish the topicality of most of the systems, since we did not contact the authors for more information. It should also be mentioned that scoping reviews do not address the risk of bias. Using only PubMed as a data source and not covering unpublished literature can have an influence on the completeness of the search. Furthermore, the study selection and data charting were only performed by one author, although they were approved by all authors. However, addressing a high methodological standard with PRISMA-ScR helped us minimize a possible bias across the study.

As this review is intended to give a broad overview of CDSSs for RDs, especially which CDSSs are available and can be used, our work is limited to collecting data about the usability of the CDSSs and effectivity of the RDs diagnosis. Some publications addressed their effectivity, but none addressed their usability in the clinical settings. We consider it necessary to involve users in the development of CDSSs, especially for clinical prototypes [40, 58–60]. We recommend to use a User-Centered Design Process (UCD), which defines an iterative process to include user requirements, needs and limitations, develop designs, prototypes and evaluate and refine it in several steps together with the users [61]. Several studies stated the importance of user-centered design in the clinical context, especially in CDSSs, to have an impact on usability and effectiveness of the systems [59, 62–64]. The use of a UCD process can help to ensure that systems are used more often in clinical routine.

Furthermore, it could be of interest at what point in the diagnosis process the CDSSs are used, since the CDSSs presented here use a variety of different approaches. More studies on the diagnosis success are needed to determine how useful these CDSS are in clinical practice.

## Conclusion

The aim of this scoping review was to give an overview of the current literature of CDSSs in RDs. The study has identified several CDSS, using different functionalities and data to support the diagnosis of RDs (e.g. analysis and comparison of phenotypic or genetic data). We have noticed that most of the CDSS are fully developed systems, which means, that they can be downloaded or used online by the clinicians. Most of the CDSS can be used for all possible diseases.

However, several improvements in the systems are useful. For instance, studies should focus on data integration to allow automatic data transfer from other systems like EHR. CDSSs developers should provide regular updates to keep the knowledge base of their CDSSs up to date.

In summary, this study shows an important overview of which CDSSs are available, by including clinical prototypes but also full developed systems, which should be interesting for developers of new CDSSs and clinicians. In the end, clinicians have to decide which system can be used for which purpose and at what stage of the diagnosis process, based on their experience and the respective patient case. Looking ahead, it remains interesting which of the CDSSs will be further developed and actively used. It is also considered important to involve clinicians in the development of the CDSSs and investigate the diagnostic success and clinical usage of CDSS in further studies.

## Supplementary information

**Additional file 1.** PRISMA-ScR checklist.

**Additional file 2.** Search process.

**Additional file 3.** Screening forms.

**Additional file 4.** Data charting form.

**Additional file 5.** Final relevant publications.

**Additional file 6.** Overview of REST-APIs.

## Data Availability

All data generated or analyzed during this study are included in this published article [and its supplementary information files].

## Abbreviations

API: Application Programming Interface
CDSS: Clinical Decision Support System
CPU: Central Processing Unit
CTX: Cerebrotendinous xanthomatosis
EHR: Electronic Health Record
ETL: Extract, Transform, Load
EU: European Union
FHIR: Fast Healthcare Interoperability Resources
GARD: Genetic and Rare Diseases Information Center
GPU: Graphics Processing Unit
HPO: Human Phenotype Ontology
ICD: International Statistical Classification of Diseases and Related Health Problems
IR: Information retrieval
MeSH: Medical Subject Heading
ML: Machine learning
MME: Matchmaker Exchange Project
NLP: Natural Language Processing
OBO: Open Biological Ontologies
OMIM: Online Mendelian Inheritance in Man
PRISMA-ScR: Prefered Items for Systematic Reviews and Meta Analyses extension for Scoping Reviews
RD: Rare Disease
REST: Representational State Transfer
UMLS: Unified Medical Language System
VCF: Variant Call Format
VSM: Vector Space Model
WES: Whole-exome Sequencing
WGS: Whole-Genome Sequencing
WHO: World Health Organization
XML: Extensible Markup Language
