# Supplementary material for: Diagnosis of Rare Diseases: a scoping review of clinical decision support systems
Source: Orphanet J Rare Dis. 2020 Sep 24;15:263. doi: 10.1186/s13023-020-01536-z (PMC7513302; doi:10.1186/s13023-020-01536-z)
Supplement: Supplementary file 2 — Additional file 2. Search process. [file 13023_2020_1536_MOESM2_ESM.pdf]

Additional file 2 – Search process

Part A: Publications with its selected keywords of the initial search

Table 1: Overview of publications of initial search

Publication title	Reference	Keywords
Utilization of Electronic Medical Records and Biomedical Literature to Support the Diagnosis of Rare Diseases Using Data Fusion and Collaborative Filtering Approaches.	Shen et al.	electronic medical record; literature; rare diseases
Can prediction models in primary care enable earlier diagnosis of rare rheumatic diseases?	Pearce et al.	Decision Support Systems, Clinical (MeSH); Decision Support Techniques (MeSH); Rare Diseases/diagnosis (MeSH)
Computer-assisted diagnosis of rare diseases	Mueller et al.	Decision Support Systems, Clinical (MeSH); Diagnosis, Computer-Assisted (MeSH); Rare Diseases/diagnosis (MeSH); Search Engine (MeSH)
Rare disease diagnosis: A review of web search, social media and large-scale data-mining approaches.	Svenstrup et al.	clinical diagnosis decision support systems; data mining; information retrieval; machine learning; rare diseases; search engines
Real time decision support system for diagnosis of rare cancers, trained in parallel, on a graphics processing unit.	Sidiropoulos et al.	Databases, Factual (MeSH), Diagnosis, Computer Assisted (MeSH); Neural Networks, Computer (MeSH); Rare Diseases/diagnosis (MeSH);

Part B: Map of keywords

Figure 1: Map of keywords

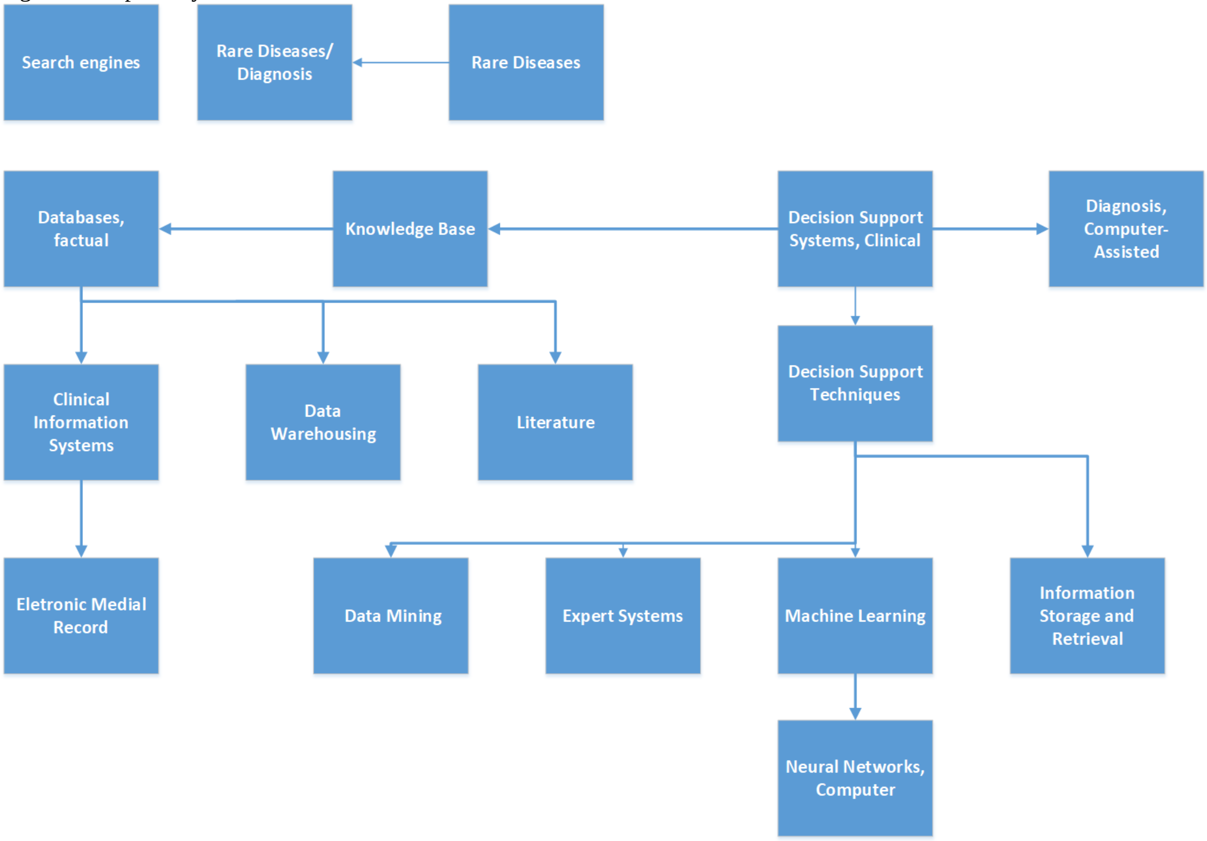

Part C: Description of the map of keywords

The term “Rare Diseases” has a relationship to “Rare Diseases/Diagnosis”. “The term “Clinical Decision Support Systems” is subdivided into “Decision Support Systems, Clinical”, which includes “Computer assisted Diagnosis” and “Clinical Decision Support Techniques”. We added the term “Knowledge base”. This is an essential term since according to our definition, it requires a knowledge base of data for decision-making. Knowledge bases can include on the one hand databases, clinical information systems like electronic medical record but also literature stored in databases. We added the term “Data Warehouse”, as a further type of a database. Furthermore, the initial search identified the term “Decision Support techniques” which can be subdivided into “Machine Learning”, “Data Mining”, “Information Storage and Retrieval” and “Neural Networks, Computer”. We further added the term “Expert Systems” as a relevant type of CDSS.

Part D: Identification of MeSH-Terms

The term **"Rare Diseases"** is a MeSH term and includes synonyms like "Orphan Diseases". The term also covers the term “Rare Diseases/Diagnosis”. The term **“Search Engine”** itself is a MeSH term. For the term Clinical Decision Support the MeSH-Term **“Decision Support Systems, Clinical”** and **“Diagnosis, computer assisted”** were used. We further identified the

MeSH terms **“Decision Support Techniques”** and **“Decision Making, Computer assisted”** as part of the MeSH-hierarchy. Furthermore, we found that the sub-terms of the MeSH term **"Decision Support Techniques"** are part of the hierarchy under the MeSH Term **"Algorithms".** This term also include the term “Expert Systems”, “Machine Learning” and “Neural Networks, Computer”. The identified term "Knowledge Base" was also part of the hierarchy of “Algorithms”. When looking at the hierarchy of MeSH, we noticed that the more general term "Database" is part of the hierarchy under the term "Information Systems" which again falls under MeSH term "Medical Informatics Applications”, which includes the MeSH term “Databases, factual”. **“Medical Informatics Applications”** also includes the MeSH term “Information Storage and retrieval” and “Data Mining”, where as “Information Storage and retrieval” include the term “Data Warehousing” as well as “Databases, Bibliographic” which covers our term Literature from above. Since all Information systems in the health care system can be relevant for Clinical Decision Support, we also used the generic term **“Medical Informatics”** for our search.

Part E: Final search terms for the PubMed search

Table 2: Final search terms

**Search Term**	**Search Group**	**MeSH-Term**	**Definition**
Rare Diseases	A	Yes	A large group of diseases, which are characterized by a low prevalence in the population. They frequently are associated with problems in diagnosis and treatment.
Search Engine	B	Yes	Software used to locate data or information stored in machine-readable form locally or at a distance such as an INTERNET site.
Decision Support Systems, Clinical	B	Yes	Computer-based information systems used to integrate clinical and patient information and provide support for decision-making in patient care.
Diagnosis, Computer-Assisted	B	Yes	Application of computer programs designed to assist the physician in solving a diagnostic problem.
Decision Support Techniques	B	Yes	Mathematical or statistical procedures used as aids in making a decision. They are frequently used in medical decision-making.
Decision Making, Computer-Assisted	B	Yes	Use of an interactive computer system designed to assist the physician or other health professional in choosing between certain relationships or variables for the purpose of making a diagnostic or therapeutic decision

Algorithms	B	Yes	A procedure consisting of a sequence of algebraic formulas and/or logical steps to calculate or determine a given task.
Medical Informatics Applications	B	Yes	Automated systems applied to the patient care process including diagnosis, therapy, and systems of communicating medical data within the health care setting.
Medical Informatics	B	Yes	The field of information science concerned with the analysis and dissemination of medical data through the application of computers to various aspects of health care and medicine.
rare diseases	C	No	No definition available (no MeSH-term).
rare disease	C	No	No definition available (no MeSH-term).
orphan diseases	C	No	No definition available (no MeSH-term).
orphan disease	C	No	No definition available (no MeSH-term).
diagnostic decision support	D	No	No definition available (no MeSH-term).

Legend: Search for Group A and B were performed with MeSH-Terms; Group C and D without MeSH-Terms
