# Supplementary material for: Diagnosis of Rare Diseases: a scoping review of clinical decision support systems
Source: Orphanet J Rare Dis. 2020 Sep 24;15:263. doi: 10.1186/s13023-020-01536-z (PMC7513302; doi:10.1186/s13023-020-01536-z)
Supplement: Supplementary file 3 — Additional file 3. Screening forms. [file 13023_2020_1536_MOESM3_ESM.pdf]

Additional file 3- Screening forms

Part A - Screening form for title and abstract screening

Table 1: Screening form for title and abstract screening

Question No	Question	Inclusion	Exclusion	Decision
1	What type of publication is the article?	• Journal Paper<br>• Conference Paper	• Literature Review<br>• Study Protocol<br>• Commentary<br>• Editorial<br>• Other	• If inclusion is true, go to question no. 2<br>• If exclusion is true, the publication is excluded
2	Is an abstract available?	Yes, an abstract is available	No, an abstract is not available	• If inclusion is true, go to question no. 3<br>• If exclusion is true, the publication is excluded
3	Is the publication written in English?	Yes, the publication is written in English	No, the publication is not written in English	• If inclusion is true, go to question no. 4<br>• If exclusion is true, the publication is excluded
4	Does the publication contain primary research or report of a Clinical Decision Support System in Rare Diseases?	Yes, the publication contain primary research or report of a Clinical Decision Support System in Rare Diseases	No, the publication contain any other description of software in Rare Diseases and not a Clinical Decision Support System	• If inclusion is true, the publication is selected for full-text screening<br>• If exclusion is true, the publication is excluded

Part B - Screening form for full-text screening

Table 2: Screening form for full-text screening

Question No	Question	Inclusion	Exclusion	Decision
1	Does the publication mainly describe a specific Clinical Decision Support System for Rare Diseases?	Yes, the publication describes a specific Clinical Decision Support System for Rare Diseases	No, the publication describes an implicit Clinical Decision Support System for Rare Diseases	• If inclusion is true, go to question no. 2.<br>• If exclusion is true, the publication is excluded
2	Does the publication describe a clinical prototype or a routine use of a Clinical Decision Support System for Rare Diseases?	Yes, the publication describes a clinical prototype or a routine use of a Clinical Decision Support System for Rare Diseases	No, the publication describes any other types of Clinical Decision Support Systems implementations (e.g. concepts, software-architectures)	• If inclusion is true, the publication is used for synthesis<br>• If exclusion is true, the publication is excluded
