# Supplementary material for: Diagnosis of Rare Diseases: a scoping review of clinical decision support systems
Source: Orphanet J Rare Dis. 2020 Sep 24;15:263. doi: 10.1186/s13023-020-01536-z (PMC7513302; doi:10.1186/s13023-020-01536-z)
Supplement: Supplementary file 4 — Additional file 4. Data charting form. [file 13023_2020_1536_MOESM4_ESM.pdf]

Additional file 4 – Data charting form

Table 1: Data charting form

Question no.	Data item	Question	Possible Answers	Multiple answers possible
1	Objective and background of the publication/project	What is the objective of the project? Why did the authors developed the CDSS?	Open answer	Yes
2	System or project name	What is the system or project name of the CDSS?	Open answer	Yes
3	Functionality	What is the functionality that performs decision support (e.g. machine learning)?	Open answer	Yes
4	Type of clinical data	Which clinical data is used for the basis of decision support?	• Clinical data<br>• Phenotypic data<br>• Genetic data<br>• Patients questionnaire<br>• Literature databases<br>• Other	Yes
5	Rare diseases covered	Which rare diseases are covered by the CDSS?	Open answer	Yes
6	Development status	What is the development status of the CDSS?	• Fully developed system<br>• Clinical prototype	No
7	System availability	Is the system available for usage?	• The system can be downloaded, no registration necessary<br>• The system can be downloaded, subject to registration	No

			• The system can be used online and free, subject to registration<br>• The system can be used online and free, no registration necessary<br>• The system is not available for personal	
8	Data entry and integration	How is data entry organized (e.g. with forms)? Can data be automatically transferred to the CDSS?	• Data entry is only possible with forms<br>• No information available<br>• Data entry with forms and data upload is possible<br>• REST-API available<br>• Data upload is possible<br>• ETL processes	Yes
9	Last software update	In which year was the last software update of the CDSS released?	• Information available (Version number, date)<br>• No information available	No
10	Current clinical usage	Are there information about the CDSS available regarding clinical usage (e.g. amount of patient cases, amount of users, participating hospitals)?	• Information available<br>• No information available	No
