# Supplementary material for: Diagnosis of Rare Diseases: a scoping review of clinical decision support systems
Source: Orphanet J Rare Dis. 2020 Sep 24;15:263. doi: 10.1186/s13023-020-01536-z (PMC7513302; doi:10.1186/s13023-020-01536-z)
Supplement: Supplementary file 5 — Additional file 5. Final relevant publications. [file 13023_2020_1536_MOESM5_ESM.pdf]

Additional file 5- Final relevant publications

Table 1: Final relevant publications

**Title**	**System or project-name**	**Functionality**	**System availability**	**Current clinical usage**	**Type of clinical data**	**Rare Diseases covered**	**Development status**	**Data entry and integration**	**Last update**
Automated semantic annotation of rare disease cases: a case study	Taboada et al.	Information Retrieval	The system can be downloaded, no registration necessary<br>Download available via URL:<br>http://www.usc.es/keam/PhenotypeAnnotation/	No information available	Literature databases	All rare diseases	Fully developed system	Data upload is possible	No information available
DECIPHER: database for the interpretation of phenotype-linked plausibly pathogenic sequence and copy-number variation	DECIPHER	Analysis or comparison of genetic and phenotypic data	The system can be used online and free, subject to registration<br>Online usage possible via URL:<br>https://decipher.sanger.ac.uk	270 centres, 36.000 patient cases	Phenotypic and genetic data	All rare diseases	Fully developed system	Data entry with forms and data upload is possible, REST-API available	No information available
Diagnostic support for selected neuromuscular diseases using answer-pattern recognition and data mining techniques: a proof of concept multicenter prospective trial	Grigull et al.	Machine Learning	The system is not available for personal use	No information available	Patients questionnaire	Neuromuscular rare diseases	Clinical prototype	No information available	No information available

Title	System or project-name	Functionality	System availability	Current clinical usage	Type of clinical data	Rare Diseases covered	Development status	Data entry and integration	Last update
Diagnostic Support for Selected Pediatric Pulmonary Diseases Using Answer-Pattern Recognition in Questionnaires Based on Combined Data Mining Applications--A Monocentric Observational Pilot Study	Rother et al.	Machine Learning	The system is not available for personal use	No information available	Patients questionnaire	Pulmonary rare diseases	Clinical prototype	No information available	No information available
Finding patients using similarity measures in a rare diseases-oriented clinical data warehouse: Dr. Warehouse and the needle in the needle stack	Garcelon et al.	Information Retrieval	The system is not available for personal use	400.000 patients included	Clinical data	• Lowe Syndrome<br>• Dystrophic Epidermolysis Bullosa<br>• Activated PI3K delta Syndrome<br>• Rett Syndrome (RETT) and Dowling Meara (EBS-DM)	Clinical prototype	ETL processes	No information available
FindZebra: a search engine for rare diseases	FindZebra	Information Retrieval	The system can be used online and free, no registration necessary<br>Online usage possible via URL:<br>http://www.findzebra.com	No information available	Literature databases	All rare diseases	Fully developed system	Data entry is only possible with forms	No information available

Title	System or project-name	Functionality	System availability	Current clinical usage	Type of clinical data	Rare Diseases covered	Development status	Data entry and integration	Last update
GeneMatcher: a matching tool for connecting investigators with an interest in the same gene	GeneMatcher	Analysis or comparison of genetic and phenotypic data	The system can be used online and free, subject to registration<br>Online usage possible via URL:<br>https://genematcher.org	8807 users from 90 countries	Phenotypic and genetic data	All rare diseases	Fully developed system	Data entry is possible with forms, REST-API available	No information available
GeneYenta: a phenotype-based rare disease case matching tool based on online dating algorithms for the acceleration of exome interpretation.	GeneYenta	Analysis or comparison of genetic and phenotypic data	The system can be used online and free, subject to registration<br>Online usage possible via URL:<br>https://geneyenta.com	No information available	Phenotypic data	All rare diseases	Fully developed system	Data entry is only possible with forms	No information available
GenIO: a phenotype-genotype analysis web server for clinical genomics of rare diseases	GenIO	Analysis or comparison of genetic and phenotypic data	The system can be used online and free, no registration necessary<br>Online usage possible via URL:<br>https://bioinformatics.ibioba-mpsp-conicet.gov.ar/GenIO/index.php	No information available	Phenotypic, genetic and clinical data	All rare diseases	Fully developed system	Data entry with forms and data upload is possible	Last update: version 1.0, 22nd of November 2017
The Matchmaker Exchange: a platform for rare disease gene discovery	Matchmaker Exchange	Analysis or comparison of genetic and phenotypic data	The system can be used online and free, subject to registration<br>Further information to connect an existing to	Information about connected platforms are available on the website	Phenotypic, genetic and clinical data	All rare diseases	Fully developed system	REST-API available	Last update: version 1.1, 20th of August 2019

Title	System or project-name	Functionality	System availability	Current clinical usage	Type of clinical data	Rare Diseases covered	Development status	Data entry and integration	Last update
			MatchMaker API: https://www.matchmakerexchange.org/i_have_a_database.html						
Real time decision support system for diagnosis of rare cancers, trained in parallel, on a graphics processing unit	Sidiropoulos et al.	Machine Learning	The system is not available for personal use	No information available	Clinical data	Rare brain cancer diseases	Clinical prototype	No information available	No information available
Utilization of Electronic Medical Records and Biomedical Literature to Support the Diagnosis of Rare Diseases Using Data Fusion and Collaborative Filtering Approaches	Shen et al.	Information Retrieval	The system is not available for personal use	No information available	Literature databases and clinical data	All rare diseases	Clinical prototype	No information available	No information available
PhenomeCentral: a portal for phenotypic and genotypic matchmaking of patients with rare genetic diseases	PhenonomeCentral	Analysis or comparison of genetic and phenotypic data	The system can be used online and free, subject to registration<br>Online usage possible via URL:<br>https://www.phenomecentral.org/	10.000 patient cases included	Phenotypic, genetic and clinical data	All rare diseases	Fully developed system	Data entry with forms and data upload is possible, REST-API available	Last update: version 1.2.0, 14th of August 2019

Title	System or project-name	Functionality	System availability	Current clinical usage	Type of clinical data	Rare Diseases covered	Development status	Data entry and integration	Last update
Phenopolis: an open platform for harmonization and analysis of genetic and phenotypic data	Phenopolis	Analysis or comparison of genetic and phenotypic data	The system can be downloaded, subject to registration<br>Online usage possible via URL:<br>https://phenopolis.org/	No information available	Phenotypic and genetic data	All rare diseases	Fully developed system	Data entry is only possible with forms	Last update: version 1.0.2, 12th of November 2017
PhenoTips: patient phenotyping software for clinical and research use.	PhenoTips	Analysis or comparison of genetic and phenotypic data	The system can be downloaded, subject to registration<br>Download available via URL:<br>https://phenotips.org/	Used in over 60 countries	Phenotypic, genetic and clinical data	All rare diseases	Fully developed system	Data entry with forms and data upload is possible, REST-API available	Last update: version 1.4.7, 17h of May 2019
Clinical Diagnostics in Human Genetics with Semantic Similarity Searches in Ontologies	Phenomizer	Analysis or comparison of genetic and phenotypic data	The system can be used online and free, no registration necessary<br>Online usage possible via URL:<br>http://compbio.charite.de/phenomizer/	No information available	Phenotypic data	All rare diseases	Fully developed system	Data entry is only possible with forms	No information available
PhenoDB: A New Web-Based Tool for the Collection, Storage, and Analysis of Phenotypic Features	PhenoDB	Analysis or comparison of genetic and phenotypic data	The system can be downloaded, subject to registration<br>Download available via URL:<br>https://phenodb.org/downloads/	No information available	Phenotypic and genetic data	All rare diseases	Fully developed system	Data entry with forms and data upload is possible	No information available

Title	System or project-name	Functionality	System availability	Current clinical usage	Type of clinical data	Rare Diseases covered	Development status	Data entry and integration	Last update
GEMINI: Integrative Exploration of Genetic Variation and Genome Annotations	GEMINI	Analysis or comparison of genetic and phenotypic data	The system can be downloaded, subject to registration<br>Download available via URL: https://gemini.readthedocs.io/en/latest/	No information available	Phenotypic, genetic and clinical data	All rare diseases	Fully developed system	Data upload and REST-API available	Last update: version 0.20.1, no date available
GenomeConnect: matchmaking between patients, clinical laboratories and researchers to improve genomic knowledge	GenomeConnect	Analysis or comparison of genetic and phenotypic data	The system can be used online and free, subject to registration<br>Online usage possible via URL:<br>https://www.genomeconnect.org/	No information available	Phenotypic and genetic data	All rare diseases	Fully developed system	Data entry with forms and data upload is possible	No information available
