# Supplementary material for: Diagnosis of Rare Diseases: a scoping review of clinical decision support systems
Source: Orphanet J Rare Dis. 2020 Sep 24;15:263. doi: 10.1186/s13023-020-01536-z (PMC7513302; doi:10.1186/s13023-020-01536-z)
Supplement: Supplementary file 6 — Additional file 6. Overview of REST-APIs. [file 13023_2020_1536_MOESM6_ESM.pdf]

Additional file 6 - Overview of REST-APIs

Table 1: Overview of REST-APIs

**CDSS**	**URL**
DECIPHER	https://release.decipherinc.com/s/local/api.html
GeneMatcher	https://genematcher.org/help/api
Matchmaker Exchange	https://github.com/ga4gh/mme-apis
PhenomeCentral	https://phenomecentral.org/download/PhenomeCentral/RemoteMatchAPI/MatchmakerExchangeremotesearchAPIv1.pdf
PhenoTips	https://phenotips.org/DevGuide/RESTfulAPI
